# Sign of Death

**Published:** 1881-05

**Authors:** 


### SIGN OF DEATH.

Dr. Chiappoli stated that he has frequently noticed in patients, apparently far from death, an extraordinary opening of the eyelids so as to give the eyes the appearance of protruding from their orbits, which was invariably a sign that death would occur within 24 hours. In some cases, when only one eye is wide open while the other remains normal, death will not follow quite so rapidly, but it does take place in a week or so. It is easy to observe this phenomenon when the eyes are wide open, but when, as is generally the case. the eyes are half shut and only open from time to time, it will be advisable to fix the attention of the patient on some point of light in order to see the change. Chiappoli is utterly at a loss to explain this symptom of approaching dissolution, and ascribes it to some diseased state of the sympathetic nerve.

